# A proposed framework for practical multimodal management of osteoarthritis in adult dogs

**DOI:** 10.3389/fvets.2026.1864824

**Published:** 2026-10-09

**Authors:** Denis J. Marcellin-Little, Janice Huntingford, Tamara Grubb, Arielle Pechette Markley, Matthew W. Brunke, Donald A. Hulse, Bethany Frank

**Affiliations:** 1 Department of Surgical and Radiological Sciences, Weill School of Veterinary Medicine, University of California, Davis, Davis, CA, United States; 2 Essex Animal Hospital, Essex, ON, Canada; 3 College of Veterinary Medicine, Washington State University, Pullman, WA, United States; 4 Red Sage Integrative Veterinary Partners, Fort Collins, CO, United States; 5 College of Veterinary Medicine, Cornell University, Ithaca, NY, United States; 6 College of Veterinary Medicine and Biomedical Sciences, Texas A&M University, College Station, TX, United States; 7 Vetoquinol USA, Fort Worth, TX, United States

**Keywords:** dog, osteoarthritis, treatment guidelines, weight management, non-steroidal anti-inflammatory drugs, nutritional supplements, nutraceuticals, physical rehabilitation

## Abstract

Osteoarthritis (OA) affects nearly half of adult dogs and has physiological, physical, and functional consequences. The physiological impact of OA includes joint inflammation, progressive cartilage degeneration, and pain. Physically, OA changes commonly result in reduced limb strength, muscle atrophy, and, in some cases, decreased joint range of motion. Functionally, OA manifests as diminished willingness or ability to climb stairs, jump, play, exercise, or perform routine activities. These functional losses negatively impact quality of life for both dogs and their caregivers. Effective OA management requires a comprehensive and multimodal approach. Therapeutic options include nutrition, nutritional supplementation, medications, lifestyle changes, electrophysical modalities, manual therapy, therapeutic exercise, ergonomic support, and surgery. Because OA is a chronic and progressive condition, management strategies must be practical, individualized, and sustainable over the long term. Protocols should be streamlined to minimize caregiver burden while maximizing clinical benefit. Foundational management includes nutritional strategies to optimize metabolic health and maintain ideal body condition. Nutritional supplements may provide safe and practical adjunctive support to decrease joint inflammation and pain. When joint pain is identified, non-steroidal anti-inflammatory drugs remain a predictably effective first-line pharmacologic therapy. Additional or alternative therapeutic interventions should be added when initial therapy is inadequate or when safety considerations limit use. Sustained and structured client communication is critical to setting expectations, monitoring response to therapy, and adapting management plans over time. Successful OA management depends not only on therapeutic selection but also on long-term adherence, reassessment, and individualized adjustment. A consensus-driven, multimodal framework provides clinicians with a practical and sustainable strategy to optimize outcomes for adult dogs with OA.

## Introduction

Osteoarthritis (OA) is the most common chronic orthopedic condition in dogs. It typically results from developmental orthopedic diseases, most commonly hip dysplasia, elbow dysplasia, and patellar luxation (1). OA may also develop as a consequence of acquired conditions, most commonly cranial cruciate ligament injury (1). Less frequently, OA may develop from other developmental orthopedic diseases (osteochondritis dissecans, angular limb deformities, developmental joint instability), trauma (e.g., articular fractures), and systemic diseases affecting joints (e.g., immune-mediated joint disease, von Willebrand disease). OA develops more rapidly in larger dogs and in dogs with excess body weight. Although OA often develops early in life and may be detected and managed in growing dogs (2), it is more frequently diagnosed and managed in adulthood. While OA and pain management in dogs have been extensively reviewed (3, 4), limited attention has been given to how life stages influence management decisions. This article is the second in a series of articles reviewing the management of OA across life stages. The previous article reviewed OA management in growing dogs (2). This article reviews the current literature and discusses OA management considerations in adult dogs. Recommendations are provided for healthy dogs at low and high risk of OA as well as dogs with clinical OA. Healthy adult dogs without underlying musculoskeletal conformational abnormalities, that are not engaged in high-intensity work or sport, and that do not belong to breeds predisposed to early-onset OA are considered at lower risk for developing OA. Because the present article references the first article in the series (2), review of that article is recommended prior to reading this manuscript.

## OA pathophysiology

OA is generally progressive; over time new clinical signs develop and existing signs gradually increase in severity and duration (5, 6). The primary consequences of OA are local physiologic changes that negatively affect joint surfaces, the joint capsule, and the subchondral bone. Articular surfaces undergo degeneration, including fibrillation and abrasion. Focal full-thickness cartilage erosion is common on the femoral head and dorsal acetabular rim of the dysplastic hip and on the medial coronoid process and medial aspect of the humeral condyle of the dysplastic elbow. The joint capsule undergoes fibrotic thickening. Osteophytes form at the margins of the articular surfaces and enthesophytes develop at intra-articular insertion sites. Inflammation persists in the joint capsule and subchondral bone (7). The extent of articular surface damage, capsular fibrosis, osteophytic proliferation, and subchondral inflammation varies widely among dogs, even within a single orthopedic condition (8–11). For example, with hip dysplasia large osteophytes develop in some joints but not others (9). Osteophyte formation in the dysplastic hip progresses more rapidly in younger dogs and in spayed females (8). Loss of joint motion secondary to capsular fibrosis and potential periarticular adhesions also varies widely (9). Severe restriction of joint motion in joints with OA may result in markedly compromised limb use and gait. The severity of inflammation associated with OA likely contributes substantially to persistent pain and disability (10).

Subjectively, adult dogs with OA often present for care with specific profiles: limb disuse associated with severe inflammation or functional decline secondary to chronic pain, muscle atrophy, and reduced joint motion. Dogs with severe inflammation are often young adults. In these cases, inflammation may persist from early disease or be triggered by excessive activity, a phenomenon described as “weekend warrior syndrome,” in which generally inactive dogs engage in strenuous exercise and subsequently exhibit OA signs. Dogs with progressive loss of function and impaired limb use are more often middle-aged to older adults. Their signs develop gradually. Their owners commonly describe them as less happy and less active. Estimating the relative contributions of inflammation, chronic pain, muscle atrophy, and reduced joint motion in an adult dog with OA can guide selection of the most appropriate management strategies. Although dogs and humans share many similarities in OA (12), structural changes are often more pronounced and progression more rapid in dogs than humans (13, 14). These differences should be considered when extrapolating evidence from human OA management.

The clinical expression of pain in canine OA also varies widely, ranging from subtle signs (minor weight shifts, mild or intermittent lameness) to severe, debilitating pain (limb disuse, non-weight bearing lameness) (9). Variability in pain expression has been attributed to local nociceptive amplification and central sensitization, as well as to neuropathic and psychogenic pain components. Less is known in dogs than humans regarding the relative contribution of these various mechanisms (15). In the absence of definitive information about the roles of nociceptive, neuropathic, and psychogenic pain, clinicians should rely on careful clinical assessment. Severe clinical signs suggest the involvement of at least one or more of these pain mechanisms and warrant comprehensive, multimodal pain management.

## Diagnosis of OA

Because the impact of OA varies widely among dogs, both with each cause of OA and across various causes of OA, it is critical to establish a physical and functional baseline at the time of initial presentation. Detection of OA is often prompted by owner concerns regarding gait or mobility, but it may also result from orthopedic screening, particularly in dogs intended for breeding, sport, or work. In some cases, OA is identified incidentally, such as when hip OA is noted on abdominal radiographs.

A baseline diagnosis of OA should identify the affected joint or joints and characterize the severity of the problem in each affected joint. Assessment of severity includes the evaluation of pain (response to palpation, limb disuse), physical changes (loss of joint motion, muscle atrophy), and functional impairment (reduced willingness to walk, play, or perform routine activities). Accordingly, diagnosis should include a thorough orthopedic examination to estimate pain severity and structural change, along with at least one validated owner questionnaire (16) to assess functional impact. Several validated instruments are available, including the Canine Brief Pain Inventory (CBPI, 2007), the Helsinki Chronic Pain Index (HCPI, 2009), the Liverpool Osteoarthritis in Dogs score (LOAD, 2009), and the Canine Orthopedic Index (COI, 2014) (16). Minimal clinically important differences have been reported for the COI (14 points) and LOAD (4 points) (17). The CBPI appears to be the most widely used client-reported outcome measure. Incorporating a questionnaire is particularly important because fewer than half of dogs with OA exhibit lameness readily recognized by owners (14, 18).

While the orthopedic examination may raise strong suspicion for OA, confirmation typically requires diagnostic imaging, as in growing dogs (2). Radiography is most often used to confirm OA based on the identification of osteophytes and enthesophytes (8). Computed tomography (CT) scans may be indicated when superimposition limits radiographic sensitivity, particularly in the elbow joint, where radiographs have limited sensitivity for detection of fragmentation of the medial coronoid process (2). CT may also be useful in the tarsus, carpus, and other complex joints and can be used to quantify OA severity (19). Overall, the correlation between imaging findings and clinical severity is weak. In one study of dogs with elbow OA, the correlation between clinically detectable pain and radiographic OA severity was poor, whereas correlation between pain and tissue inflammation identified using positron emission tomography scanning was stronger (10). Arthroscopy can also be used to diagnose OA and can combine the diagnostic evaluation with a therapeutic intervention. For example, elbow arthroscopy can confirm OA and allow for removal of a fragmented medial coronoid process. Small diameter (needle) scopes reduce invasiveness (20), although their capacity for concurrent treatment is more limited than that of conventional rigid arthroscopes. Accurate diagnosis is particularly important in sporting and working dogs, in whom OA-associated pain may adversely affect training and performance. Regular screening using owner questionnaires, observation, and palpation may increase the likelihood of early detection in these populations.

In summary, OA may be identified through owner concern, screening questionnaires, clinical examination, or incidental imaging findings. The severity of clinical signs does not consistently correlate with the severity of structural changes on imaging but may more closely reflect the degree of tissue inflammation within and surrounding the affected joint.

## Whole body wellness in adult dogs with OA

Client education regarding the signs of OA pain and the anticipated progression of disease is fundamental to successful long-term management. Clients should understand that, as OA progresses, clinical signs may include joint swelling, pain on palpation, decreased range of motion, stiffness, muscle atrophy, reduced limb use, and diminished mobility. Functional changes such as difficulty climbing stairs, limping, trouble rising, or inability to jump are also commonly associated with OA pain in adult dogs. Painful dogs may withdraw from interaction or respond with irritability or aggression when touched. These behavioral changes can complicate OA management and can strain relationships within the household.

As mobility declines, environmental challenges within the home become increasingly relevant. For example, a dog may struggle to climb steep stairs or navigate slippery floors. Although these challenges are often associated with geriatric dogs, they frequently begin in middle-aged adults. Recommending environmental modifications, such as rugs, mats, or carpet runners, may help improve traction and safety. Elevating food and water dishes can enhance comfort, and ramps may facilitate access more effectively than steps.

Whole body wellness supports both quality of life and long-term disease control. It encompasses intentional attention to lifestyle structure, nutrition, and exercise. Establishing a consistent daily routine can improve compliance and minimize fear, frustration, and anxiety. Baseline functional information should be collected early so that owners can identify and report meaningful changes in mobility, activity level, or demeanor that may indicate disease progression or a flare-up.

A collaborative relationship between the owner and the veterinary advisor is essential. Management decisions should reflect the owner's desired level of involvement, willingness to pursue therapies supported by varying levels of evidence, financial considerations, and preferences regarding home-based vs. clinic-delivered care. Clear, ongoing communication between the owner and patient care management team facilitates timely adjustment of management strategies and supports sustainable long-term care.

A nutritional plan should support maintenance of a lean body condition. If the dog is overweight, structured guidance, including dietary recommendations, caloric targets, and appropriate weight loss rates, should be provided to the dog owner. Nutritional supplementation may be discussed and implemented based on individual patient needs.

Exercise and play should be incorporated into the dog's daily routine, as greater activity levels are associated with fewer clinical signs of lameness (9). Activity should be individualized for both the dog and the owner. Dogs should be conditioned gradually, and owners should be trained to recognize signs of fatigue or pain. Intensity, frequency, and duration of exercise should be defined, with structured plans to improve strength and endurance when appropriate. A proactive plan should be established to modify activity type, intensity, or duration in the event of an OA flare or when increased clinical signs are consistently associated with specific exercises or workloads. Dogs should also be trained to engage in appropriate behaviors, avoiding lead pulling and other counterproductive patterns, as lead pulling appears to negatively affect the quality, duration, and frequency of walks (21). For sporting and working dogs, whole body wellness includes balancing training and performance demands with recovery. Rest must be intentionally incorporated into training schedules to prevent overuse injuries and chronic stress.

### Whole body wellness: key points

Whole body wellness is fundamental to managing OA in adult dogs. A consistent and appropriately modified lifestyle, optimized nutrition, structured exercise, and balanced work or sport participation help to maintain strength and fitness, reduce the likelihood of OA flares, and support sustained control of OA over time.

## Physical wellness in adult dogs with OA

### Body condition

Fitness refers to a dog's ability to perform functional activities efficiently and effectively. It encompasses cardiovascular and respiratory health, musculoskeletal strength, endurance, and agility. Fit dogs are expected to demonstrate greater mobility and flexibility, improved overall cardiovascular fitness, improved exercise recovery, and reduced risk of chronic diseases. Optimizing fitness promotes healthspan—the period of life spent in good health, free from chronic disease and disability (22, 23).

Aging is commonly associated with musculoskeletal change, including loss of muscle mass and generalized sarcopenia (23). Fatness (adiposity) refers to the proportion of body fat relative to lean body mass. Ideal body condition corresponds to a body condition score (BCS) of 4 or 5 out of 9 (24). Overweight dogs (BCS 6/9) carry excess adipose tissue, and obese dogs (BCS ≥ 7/9) typically exceed ideal body weight by 20% or more. Excess adiposity negatively affects overall health: overweight dogs are at an increased risk for systemic illnesses such as diabetes, cardiac disease, and OA, and have a shorter life expectancy (6, 25). They also have decreased stamina and increased susceptibility to overheating (26). As dogs age, their body fat percentage generally increases, although weight loss may be observed in very old dogs (23, 27). Fitness and fatness are inversely correlated, although a dog may be overweight yet physically conditioned or lean yet poorly conditioned. Many dogs are overweight or obese (28, 29), and owners frequently underestimate their dog's body condition; more than one-third of owners may not accurately assess BCS (28, 30). Sporting and working dogs are expected to have higher fitness levels than sedentary dogs due to structured training. In one study, mean body fat percentage was lower in sporting dogs (13.8 ± 5.3%) than in non-sporting dogs (19.1 ± 8.6%) (30). Excess body weight is clinically significant in the management of OA as it exacerbates systemic inflammation and the signs of OA in dogs (25, 31, 32).

Neutering increases the risk of weight gain (2) even when performed in adulthood (33), and is associated with an increased risk of certain orthopedic conditions, including hip and elbow dysplasia and cranial cruciate ligament injury (34–36). In one study, OA progression was more rapid in spayed females, possibly related to estrogen deficiency (8). When a dog has an ideal body condition, a collaborative nutrition plan should be implemented to maintain it. For overweight dogs, structured support should include calculation of daily energy requirements, dietary recommendations (including foods to avoid), a target weight, and a safe rate of weight loss. Nutritional supplementation should be discussed and implemented based on specific patient needs.

Canine obesity is a complex metabolic disease often present in dogs with OA. Successful weight loss regimens require a controlled negative energy balance, while preserving lean body mass (LBM) and preventing nutritional deficiencies. Therapeutic diets require an elevated protein-to-calorie ratio. Clinical guidelines recommend > 30 to 40% protein dry matter (DM) or > 85 g of protein per 1,000 kcal to support muscle maintenance during lipolysis. Restricting fat (typically to 8 to 12% DM) is the primary method for reducing overall caloric density in dog food. However, formulations must still provide adequate essential fatty acids (e.g., linoleic acid) to maintain epidermal barrier function (37). Elevated fiber (typically, 10 to 15% DM or more) dilutes energy density and promotes satiety via gastric distension and delayed emptying, which experimentally shows efficacy. However, clinically it may not provide the gastric fill required for long-term satiety. An optimal diet uses a blend of primarily insoluble fiber for bulking and lesser soluble fiber for glycemic and microbiome support (37). Restricting standard diets for weight loss is not recommended, as it risks micronutrient deficiencies. Therefore, purpose-formulated therapeutic diets are optimal, providing an elevated nutrient-to-calorie ratio to ensure 100% daily requirements for vitamins and minerals, despite caloric restriction. Many of these diets contain higher protein to preserve lean body mass (LBM) during weight loss. Also, omega-3 fatty acids can be added with the intent to modulate inflammation and support joint health. However, omega-3 concentration in many weight-loss diets is not sufficient to alleviate the inflammation associated with OA. For optimal outcomes, practitioners should calculate daily caloric targets using the patient's ideal body weight (IBW). For a weight-loss program, the calories fed to dogs is based on resting energy requirement (RER) using the formula: RER = 70 x (IBW kg)^0.75^. Weight-loss programs often start by feeding 80% of the RER corresponding to the IBW. Bi-weekly to monthly monitoring of body weight and BCS is crucial to titrate intake and ensure a safe 1 to 2% weekly weight loss. Weight maintenance programs for obesity-prone dogs should also include moderately high protein intake to preserve LBM and support a healthy basal metabolic rate. Dogs in weight maintenance programs should be fed at RER for the ideal body weight (37).

### Activity and exercise

Body composition and physical activity are closely interconnected and together influence musculoskeletal health and the clinical expression of OA in dogs. Historically, activity restriction was commonly recommended for adult dogs with OA, in part because transient increases in lameness may occur following exercise (38). However, over the long term, greater activity levels are associated with fewer clinical signs of lameness in dogs with OA (9). Similarly, exercise decreases pain and improves function in humans with knee OA (39). Exercise and play should therefore be incorporated into the dog's daily routine. Activities must be adapted to the individual patient and owner. Dogs should be trained to engage in appropriate exercise behaviors, avoiding lead pulling and other patterns that reduce comfort, safety, and effectiveness of activity (21). Baseline intensity, frequency, and duration of exercise should be established, with structured plans to improve strength or endurance when appropriate.

Exercise programs should align with the dog's functional goals, whether maintaining general fitness, engaging in recreational play, or preparing for sport or work. Owners and trainers should remain mindful of risks associated with specific activities. For example, in one study, daily hikes or runs were associated with an increased risk of cranial cruciate ligament injury in agility dogs (40). Signs of fatigue or discomfort must be recognized and addressed. A proactive plan should be developed to modify activity type, duration, or intensity during symptom flares or when increased clinical signs are consistently associated with specific workloads.

### Physical rehabilitation

In adult dogs with OA undergoing physical rehabilitation, the management plan should consider both patient and owner factors. Patient factors include age, size, temperament, training status, severity of pain and disability, and the presence of comorbidities or risk factors for symptom flares. Owner factors include goals, experience, motivation, available time, financial resources, physical ability to assist with exercises, and other practical limitations that may affect adherence to the rehabilitation plan. Rehabilitation plans consider the type, frequency, intensity, and duration of activity. Dogs with greater risk factors or more severe clinical presentations generally require a more progressive and multifaceted management program (41). Expected outcomes differ between pet dogs and working dogs. Pet dogs should ideally be comfortable during routine daily activities and play. In contrast, working dogs are expected to perform their occupational or sporting activities without functional limitations.

Pain must be minimized before initiating or intensifying an exercise program. The simplest approach capable of achieving adequate pain control is generally preferred. In some dogs, pain can be managed with medication alone. In others, effective pain control may require a combination of pharmacologic therapy, nutritional supplementation, electrophysical modalities, or manual therapy. Once pain is adequately controlled, therapeutic exercise becomes a central component of OA management. Exercise intensity and duration are gradually increased as the dog progresses toward treatment goals. Periods of rest between activities should be tailored to prevent fatigue, declines in performance, or recurrence of clinical signs. Over time, management plans often become simpler and rely increasingly on owner-delivered care, such as home exercise programs and routine activities that maintain fitness while avoiding symptom flares.

### Patient assessment

Patient assessment is essential for developing individualized treatment goals, creating an OA management plan, and selecting outcome measures to evaluate response to therapy. Assessment typically includes observation and analysis of stance and gait during walking, trotting, and selected functional activities such as stair climbing. The evaluation also includes a general physical examination, a detailed orthopedic examination, and a brief neurological examination to rule out concurrent spinal pain or ataxia. The orthopedic examination includes evaluation of joint motion and stability, measurement of range of motion using goniometry, limb girth measurements, and assessment of functional mobility and strength. Baseline assessment should include detailed documentation of both objective and subjective measures so that response to therapy and disease progression can be monitored over time. Comprehensive rehabilitation assessments are described in greater detail in other resources (41–44). Key parameters that influence the development of an OA management plan are discussed below.

### Joint range of motion (ROM)

OA can result in loss of joint motion; however, not all joints affected by OA demonstrate loss of motion and the degree of restriction varies widely among dogs (9). Range of motion (ROM) is often measured at baseline and during follow-up examinations, particularly when abnormal joint motion is observed or suspected. Adequate joint motion is necessary for dogs to stand and move without restriction. Reduced ROM can negatively affect mobility (for example, limiting the ability to climb stairs or to transition from sitting to standing), may exacerbate chronic pain, and could lead to compensatory changes in other joints, the spine, and associated musculature. Increasing joint motion is therefore an important therapeutic goal when a joint lacks the mobility required for normal daily activities.

Range of motion is typically measured using goniometry, in which a goniometer is aligned with anatomical landmarks proximal and distal to the joint while the limb is passively moved through flexion and extension to quantify the joint angle in degrees (45). The severity of motion restriction helps guide therapeutic interventions such as targeted exercises, manual stretching, and joint mobilization techniques (46). Goniometric measurements provide objective data that can be used to monitor response to therapy or detect deterioration associated with disease progression (47). Traditional goniometers are inexpensive, easy to use, and have been shown to provide greater accuracy than smartphone or digital measurement tools (48). Anatomic landmarks and normal ranges of joint motion have been described (43, 44, 47, 48). Individual dogs also differ in their standing joint angles; some exhibit more upright posture, while others maintain a more flexed or crouched stance (49).

#### Muscle mass assessment

Reduced weight bearing associated with pain, OA, and compensatory gait patterns commonly leads to muscle atrophy. Muscle girth should therefore be measured at baseline and monitored at regular intervals during treatment. Several techniques can be used to assess muscle mass, including limb girth measurements, ultrasound, CT, MRI, and dual-energy x-ray absorptiometry (DEXA) (50–52). Limb girth measurement is the most practical method in clinical settings because it is simple and inexpensive. However, measurements are influenced by the technique used and must be performed consistently to allow meaningful comparison over time (13, 50, 51, 53).

#### Functional mobility and strength assessment

In human physical therapy, functional mobility tests, strength assessments, and task-specific challenges are routinely used to establish treatment goals, design therapy programs, and provide measurable outcomes (54–59). Similar functional assessment tools have been explored for use in dogs, although their validation remains limited (43). Proposed tests for evaluating mobility in dogs include the Timed Up and Go, Cavaletti rails, Figure of 8s, and Down to Stand (43). The Timed Up and Go (TUG) test has recently been validated for use in dogs and provides a practical and objective measure of functional mobility by recording the time required for a dog to rise from a stationary position and walk a short distance (60, 61). These tests may provide useful information about functional performance and may help monitor changes in mobility over time.

#### Activity monitoring and wearable sensors

Activity monitoring has been used to assess mobility and activity patterns in dogs. In general, larger and older dogs tend to be less active than younger and smaller dogs (62). An increase in activity of approximately 20% was reported following administration of pain medication in dogs with OA in one study (63), whereas no change in activity was detected after weight loss in another study (64). Early activity monitoring systems were considered somewhat subjective due to limited accuracy (65) and uncertainty regarding the relationship between activity levels and clinical measures of mobility or pain (66). However, advances in sensor technology and data analysis have improved the performance of wearable activity monitors (67, 68). As a result, their use as objective outcome measures in clinical trials of OA treatments in dogs has increased (69, 70).

### Management options

*Therapeutic exercise* is considered the cornerstone of physical rehabilitation in dogs with OA because it directly addresses strength, mobility, and functional capacity. Exercise programs typically include both static and dynamic activities. Static exercises emphasize postural control and foundational stability, which are important for all patients regardless of their clinical patient profile, severity of OA, activity level or performance demands. One structured example is the Fit to Work program, developed by the Penn Vet Working Dog Center at the University of Pennsylvania (39). The program incorporates a fitness assessment, structured exercises, and progressive exercise advancement tailored to individual dogs.

*Walking* is the most common form of exercise in dogs and has been evaluated in several studies. Longer daily exercise duration (> 60 min/day) has been associated with a decrease in the severity of lameness in dogs with hip dysplasia compared with limited daily exercise (< 20 min/day) (9). Incline and decline walking have also been evaluated for their effects on joint motion and can be incorporated into targeted exercise programs (58, 59). Other dynamic exercises that can be used to selectively influence joint range of motion include Cavaletti poles and stair exercises (58, 59, 71–75). These exercises can be selected, modified, and progressed based on the desired joint effects (e.g., improving hip extension or elbow flexion) as well as the specific muscle groups being targeted. For each exercise, baseline frequency, intensity, and duration should be selected based on the dog's current abilities and owner factors. These parameters are modified over time, based on response to therapy and on the ability of the owner to sustain the exercise program.

*Hydrotherapy* involves the therapeutic use of water and commonly includes underwater treadmill exercise and swimming (76). Joint motion effects differ substantially between underwater treadmill therapy and swimming, which can influence the selection of therapy when specific range-of-motion or loading effects are desired. Underwater treadmill therapy allows controlled weight-bearing exercise, whereas swimming typically produces greater joint flexion with minimal limb loading. Muscle activation during underwater treadmill exercise also varies with water depth due to the interaction between limb motion and the physical properties of water. Adjusting water level can therefore influence joint loading, range of motion, and muscle recruitment during therapy (77, 78).

*Manual therapy* includes massage, stretching, joint mobilization, myofascial release, and traction. These techniques can assist with both assessment and treatment of musculoskeletal pain. Palpation of affected tissues provides valuable information about the source and severity of pain associated with joint motion or tissue compression. Passive range of motion (PROM) exercises provide non-painful sensory stimulation, may reduce swelling, and gently stretch periarticular tissues. PROM has been reported to relieve pain in some patients (79). Many owners can be trained to perform PROM exercises at home. Massage may also be beneficial to dogs with OA by providing non-noxious sensory stimulation, improving venous and lymphatic drainage, and potentially promoting endorphin and serotonin release. In humans with knee OA, manual therapy combined with exercise has been shown to provide greater benefit than electrophysical modalities combined with exercise (80). Although massage has demonstrated benefits in humans with OA (81), controlled studies evaluating manual therapy for chronic pain management in dogs remain limited (82–85).

*Photobiomodulation therapy (PBMT)*, also known as low-level laser therapy or cold laser therapy, uses light at specific wavelengths to influence cellular activity. PBMT may affect pain perception by altering peripheral and central neurochemistry, afferent nerve conduction, and endorphin release, thereby increasing pain thresholds. PBMT also produces several physiologic effects, including increased myofibroblast activity, enhanced collagen and cartilage synthesis, increased tenocyte and myocyte activity, and increased ATP production through altered mitochondrial oxygen utilization. Additional effects include improved blood flow through angiogenesis and vasodilation, as well as stimulation of axonal sprouting in the peripheral and central nervous systems (86). Clinical trials evaluating PBMT in dogs have produced mixed results, possibly due to small sample sizes and variability in treatment protocols (87, 88). Optimal dosing parameters for PBMT in dogs remain unknown.

*Pulsed electromagnetic field therapy* (PEMF) modulates the calcium-calmodulin nitric oxide cascade and downregulates IL-1β, thereby reducing inflammation (89). In humans, PEMF has demonstrated small to moderate benefits in the treatment of knee OA (90–92). In dogs, randomized controlled studies have shown reductions in postoperative pain and OA-associated pain, as well as improved neurologic recovery after thoracolumbar intervertebral disc extrusion (93, 94). PEMF devices used in veterinary medicine commonly deliver therapy through loops or treatment mats.

*Extracorporeal shockwave therapy* uses high-energy acoustic waves that release mechanical energy when they encounter changes in tissue density. Shockwave therapy stimulates the production of cytokines and growth factors, endothelial nitric oxide synthase (eNOS) and bone morphogenetic protein (BMP) expression. In veterinary medicine, ESWT has most commonly been investigated for treatment of tendon and ligament injuries and for stimulation of bone healing rather than specifically for OA management (95, 96). However, studies in humans suggest that ESWT may be beneficial for OA treatment (97–99).

*Thermal therapy—*Cold therapy decreases inflammation, histamine release, and edema. It may also decrease pain by reducing muscle spasm, lowering prostaglandin concentration, and slowing nerve conduction. Heat therapy increases blood flow, promotes muscle relaxation, and improves tissue extensibility (100–106).

*Acupuncture* modulates the activity within the peripheral, central, and autonomic nervous systems. Acupuncture alters the pain pathway through the release of neurochemicals, including enkephalins, endorphins, dynorphins, serotonin, norepinephrine, glutamate, neurokinin, and cannabinoids (107, 108). More recently, research focused on acupuncture as a neuromodulatory intervention, capable of influencing pain-related neuroplasticity (hyperalgesia/allodynia) and reducing neuroinflammation in the spinal cord (109, 110). Randomized controlled trials investigating the benefits of OA management using acupuncture show mixed results (111–113), but the body of evidence supporting the use of acupuncture in OA management is increasing as more trials are performed.

*Environmental modifications* represent an ergonomic strategy for dogs that have difficulties performing routine activities. Modifications may include improving floor traction, installing ramps, adjusting bedding, and modifying the height and location of food and water bowls. Additional supportive measures include foot care (such as nail trimming, hair trimming, and traction aids) and mobility assistance devices including harnesses and ambulation carts.

### Applying rehabilitation strategies based on OA risk

Physical rehabilitation planning integrates findings from the patient assessment with owner goals, expectations, and practical considerations (41, 74). Individualized exercise plans and activity modification form the foundation of rehabilitation programs for dogs with OA (41). Additional therapies, including manual therapy, electrophysical modalities, and environmental modifications, may be incorporated depending on the patient's needs and the available evidence supporting their use. Because the strength of evidence varies across rehabilitation interventions, preference should be given to strategies supported by robust scientific data.

#### Healthy adult dog/low OA risk

A rehabilitation evaluation may be beneficial even for dogs presenting without owner concerns, as underlying problems may otherwise remain undetected. Potential findings include mild conformational abnormalities, myofascial trigger points, muscle tension, reduced flexibility, or muscle imbalance (114). When risk factors for OA are minimal, rehabilitation planning primarily focuses on maintaining fitness, lean body mass, and musculoskeletal strength. Exercise programs typically emphasize general physical conditioning and are tailored to the dog's routine activities, sport participation, or working role.

#### Healthy adult dog/high OA risk

Dogs at increased risk of OA benefit from periodic reassessment to detect early changes in gait, posture, mobility, or pain responses to palpation. In addition to maintaining general fitness, exercise programs may target strengthening of joints that are predisposed to OA. For example, dogs with hip laxity that are at high risk of developing hip OA may benefit from exercises designed to strengthen the gluteal and thigh musculature with the aim to improve joint stability.

#### Adult dog with clinical OA

In dogs with established OA, rehabilitation planning is guided by the clinical assessment. Affected dogs commonly exhibit altered posture or gait, pain on palpation, reduced joint motion due to capsular thickening, muscle atrophy, and compromised mobility. Initial rehabilitation goals focus on minimizing pain during activity and restoring functional mobility through targeted exercises (41). Selection of therapeutic interventions is based on the rehabilitation assessment, owner expectations, and available evidence supporting treatment efficacy. Environmental modifications may also be implemented when mobility limitations interfere with daily activities.

### Physical wellness: key points

Physical wellness is a central component of OA management in dogs. Maintaining fitness and mobility begins with a baseline evaluation followed by exercise programs tailored to the individual patient and owner. Regular daily activity is one of the most effective strategies for preserving musculoskeletal health. Careful control of exercise intensity and duration helps reduce the likelihood of symptom flares.

## Nutritional supplementation for adult dogs

Diet and nutritional supplements (nutraceuticals) recommended for adult dogs with OA aim to reduce inflammation, improve function, and potentially slow disease progression (2). Nutritional supplementation generally has an excellent safety profile. As in growing dogs with OA, nutritional supplements are commonly incorporated into the overall health management of adult dogs with OA (2).

### Healthy adult dog/low OA risk

Adult dogs at low risk of developing OA should maintain an optimal body condition. In these dogs, nutritional supplementation is most often considered in the context of training or engaging in strenuous sporting or working activities. In these situations, supplements may be used to support musculoskeletal health, enhance performance capacity, or potentially protect joints from activity-related stress.

### Healthy adult dog/high OA risk

Healthy adult dogs with musculoskeletal conformational abnormalities, dogs that engage in high-intensity work or sport, and dogs from breeds predisposed to early-onset OA are considered at higher risk for developing OA, particularly since some strenuous activities increase the risk of injury. Maintaining optimal body weight is particularly important in these patients. The supplements recommended for adult dogs with OA are generally similar to those recommended for growing dogs with OA and include omega-3 fatty acids, undenatured type II collagen, glucosamine and chondroitin (2). Most of the available scientific evidence supporting nutritional supplementation has been generated in studies involving adult dogs (3).

### Adult dog with clinical OA

Nutritional supplementation for adult dogs with clinical OA typically includes the same supplements recommended for high-risk dogs including omega-3 fatty acids, undenatured type II collagen, glucosamine and chondroitin (2). Comorbidities such as obesity and chronic renal disease are relatively common in adult dogs with OA. The presence of renal or gastrointestinal comorbidities may increase the risk of adverse events associated with pharmacologic pain management, which may increase the appeal of nutritional supplementation as an adjunct or alternative to non-steroidal anti-inflammatory drugs (NSAIDs). Nutritional supplementation has been associated with increased weight bearing in affected limbs and a reduced perceived need for pain medications such as NSAIDs in some studies (3, 115).

### Omega-3 fatty acids

Omega-3 fatty acids are a key nutritional supplement used to reduce joint inflammation. Eicosapentaenoic acid (EPA) promotes the production of eicosanoids that are less pro-inflammatory than the byproducts of arachidonic acid catabolism (2). Supplements derived from marine sources of omega-3 fatty acids are generally more effective than short-chain plant sources such as flax seed oil (116). In one study, a diet containing 3.5% omega-3 fatty acids from fish oil decreased pain and lameness, improved weight bearing, and reduced the need for NSAIDs in dogs with OA (117). Achieving similar omega-3 concentrations through supplementation alone would require approximately 480 mg/kg of fish oil (50 to 100 mg/kg EPA), which may be costly and impractical in some cases. Green-lipped mussel extracts provide an alternate source of omega-3 fatty acids and also contain glycosaminoglycans and chondroitin sulfate. These extracts are lower in calories because the active compounds are more concentrated (118). Supplementation with green-lipped mussel extracts has been shown to benefit dogs with OA (119) and may enhance the effectiveness of NSAIDs (118).

### Undenatured collagen type II (UC-II®)

Supplementation with undenatured type II collagen is based on the principle that sustained oral administration increases the immune tolerance to collagen byproducts present in joints with OA (3). UC-II, a patented form of undenatured type II collagen derived from chicken sternum, has demonstrated safety and efficacy in several clinical studies in dogs (3). In a prospective controlled clinical trial, undenatured type II collagen supplementation decreased inflammation, cartilage catabolism, and soreness following strenuous activity in healthy field-trial dogs (120, 121). In dogs with OA, undenatured type II collagen supplementation has been associated with objective improvements in limb use measured using force-plate gait analysis in multiple studies (122–125). In one clinical trial comparing undenatured type II collagen supplementation with NSAIDs in dogs with OA, both treatments produced similar benefits (115, 126).

### Glucosamine/chondroitin supplements

Glucosamine and chondroitin sulfate (GCS) supplements are among the most used joint supplements in dogs. While the anti-inflammatory activity has been demonstrated (127), clinical efficacy of GCS supplements in dogs with OA has been inconsistent: some clinical trials report improvement (128), whereas others do not demonstrate significant benefits (129, 130). These mixed findings have led some authors to conclude that glucosamine and chondroitin provide limited therapeutic benefit (3).

### Eggshell membrane supplementation

Eggshell membrane (ESM) extracts are used as nutritional supplements and contain collagen, glucosamine, glycosaminoglycans, elastin, hyaluronic acid, and various amino acids and proteins. Many formulations combine ESM with additional compounds such as astaxanthin, krill oil, and hyaluronic acid. *In vitro* studies suggest that ESM may modulate the inflammatory pathways and potentially reduce cartilage degradation. The scientific evidence supporting eggshell membrane use in OA management is limited overall. Two pilot studies in Labrador retrievers demonstrated decreased concentrations of the inflammatory biomarker interleukin-6 (IL-6) following ESM supplementation; however, LOAD scores did not improve (131, 132). In a 90-day controlled clinical trial, ESM supplementation resulted in improved CBPI and LOAD scores (133).

### Supplementation to manage the potential loss of muscle mass

Sarcopenia refers to the gradual loss of muscle mass, quality, and strength that occurs with aging. Muscle loss associated with cachexia or sarcopenia involves increased myocyte apoptosis and reduced regenerative capacity (134). In humans, sarcopenia is associated with frailty, increased risk of physical disability, reduced quality of life, and mortality (135). Pro-inflammatory cytokines including IL-6, IL-1β, transforming growth factor-β (TGF-β), and tumor necrosis factor-α (TNF-α) contribute to muscle fiber breakdown (136). Information regarding sarcopenia in dogs with chronic OA is limited. In one study of Labrador retrievers 8 years old and older, sarcopenia in the absence of an increase in inflammatory mediators was a predictor of early mortality (137). Clinically, OA-associated limb disuse is widely believed to contribute to muscle atrophy in dogs. Because preservation of muscle mass is closely associated with maintenance of physical function in humans (135), strategies that support muscle mass may also benefit dogs with OA.

Fortetropin^®^ is a nonthermal-pasteurized, freeze-dried, fertilized egg yolk product used to support muscle mass in dogs. Myostatin, a myokine belonging to the TGF-β family, regulates muscle growth through effects on activin receptors (ACVR2A/B) (135). Fortetropin is believed to promote muscle growth by reducing circulating myostatin concentrations. Several studies in dogs demonstrate an inverse relationship between myostatin expression and muscle mass (138). In one trial involving dogs with OA, improvement in LOAD scores was observed between 6 and 12 weeks of supplementation, although differences from placebo were not statistically significant (137). In another study evaluating muscle atrophy following stifle surgery, dogs receiving Fortetropin experienced less postoperative muscle loss than the controls (139).

Ursolyx™ contains ursolic acid which is a plant-derived pentacyclic triterpene compound with anti-inflammatory, antioxidant, anti-carcinogenic, anti-obesity, and thermogenic properties (136). Mechanistically, ursolic acid influences gene expression in skeletal muscle cells, supporting cellular pathways involved in maintaining muscle structure and function (140). In dogs, dietary supplementation with ursolic acid has been associated with increased skeletal muscle mass, muscle quality, strength, and endurance (140). In one study on age-related muscle atrophy, ursolic acid supplementation suppressed more than 50 molecular markers associated with muscle loss while activating markers associated with muscle health and exercise capacity (140).

### Cannabis supplementation

The main phytocannabinoids used as supplements are cannabidiol (CBD) and Δ9-tetrahydrocannabinol (Δ9-THC). Based on the presence of a range of receptors involved in inflammation and analgesia in the pain pathway that CBD interacts with, CBD likely plays a role in analgesia. In one study, a CBD product was used safely to manage clinical signs of OA in 22 dogs (141). Because cannabis-derived compounds are regulated as drugs rather than nutritional supplements in many jurisdictions, local regulatory requirements should be reviewed before recommending, prescribing, or dispensing cannabis nutraceutical supplements.

While several clinical studies using phytocannabinoids have been published (141–145), no study used pharmaceutical-grade CBD. Therefore, little is known about the clinical safety and efficacy of pharmaceutical-grade CBD in dogs. The use of CBD-rich hemp nutraceuticals appears promising in dogs. CBD-rich hemp has been used in combination with non-steroidal anti-inflammatory drugs (NSAIDs), prednisone, amitriptyline, and gabapentin, without any serious adverse events in short-term studies. This suggests that pharmaceutical-grade CBD could be beneficial to multimodal pain management. CBD-rich hemp has also been used as a single agent (141, 142) and appears to be effective for the management of OA pain in dogs. Differences in dosing among studies may have resulted from differences among products used. In the absence of FDA regulation, the efficacy and safety of CBD-rich hemp cannot be confirmed. Clinicians can select CBD supplements with positive clinical trial evidence in dogs.

### Nutritional supplements: key points

Most nutritional supplements have a favorable safety profile. They appear most effective at reducing inflammatory pain associated with chronic OA. Some supplements may provide additional benefits not addressed by other therapies, such as improving the tolerance to collagen degradation byproducts (undenatured type II collagen) or supporting preservation of muscle mass (fortetropin, ursolic acid). Overall, information about the size of the effects of nutraceuticals used in chronic OA in dogs is lacking. When managing OA in humans, glucosamine-chondroitin and undenatured type II collagen have mild-to-moderate effects to relieve signs of OA and some herbal formulations have large and clinically significant effects (146).

## Pharmaceutical management of OA in adult dogs

Medications are the most widely used strategy to alleviate the pain signs of OA in dogs, likely because of their predictable efficacy, convenience of administration, generally acceptable safety profiles, and relatively low cost. The drugs used to manage OA in dogs belong to several pharmacologic classes. The pharmacologic treatment options for adult dogs with OA are shown in Table 1. Most pharmacologic agents act by reducing inflammation or modulating pain-related neurotransmission, although some agents target joint structure or cartilage metabolism, such as polysulfated glycosaminoglycans. The strength of scientific evidence supporting the efficacy of these drugs varies widely. NSAIDs have robust evidence supporting their ability to relieve pain and improve function, whereas other drug classes have limited or inconsistent evidence. Safety profiles also vary. Although adverse drug events are uncommon overall, they can range from mild and transient effects (e.g., a single episode of vomiting) to severe complications, including life-threatening illness or death (147).

**Table 1:** Treatment options, including pharmacologic options, for adult dogs with OA.

OA stage	Drugs	Rationale
Healthy adult dog/low OA risk	None needed	± Weight and nutritional optimization
Healthy adult dog/high OA risk	Likely none needed	Weight and nutritional optimization/supplementation
Adult dog with clinical OA	Mild pain	NSAIDs Weight and nutritional optimization/supplementation
	Moderate pain	NSAIDs or antiNGF mAbs, if tolerated ± Gabapentinoid or/and NMDA receptor antagonist Weight and nutritional optimization/supplementation ± Environmental modifications ± Intraarticular therapy
	Severe pain	NSAIDs and/or antiNGF mAbs, if tolerated in the long term Gabapentinoid or/and NMDA receptor antagonist Weight and nutritional optimization/supplementation Environmental modifications ± Intraarticular therapy

NSAIDs, non-steroidal anti-inflammatory drugs; antiNGF mAbs, anti-nerve growth factor monoclonal antibodies; NMDA, *N*-methyl-D-aspartate.

### Non-steroidal anti-inflammatory drugs

NSAIDs are potent analgesics that primarily reduce pain by inhibiting prostaglandin E_2_ (PGE_2_) production. Several NSAIDs are FDA-approved for both acute and chronic pain management in dogs. Because of their predictable efficacy in most dogs with OA, NSAIDs are widely considered the cornerstone of pharmacologic management of OA (3, 148, 149). Detailed reviews of NSAID use in dogs are available elsewhere (150). Both veterinarians and owners observe improvement in most dogs receiving NSAIDs. In one study of 1,000 dogs treated with an NSAID for 40 days, two-thirds of dogs improved within 14 days and three-quarters improved by 40 days (151). Most NSAIDs inhibit cyclooxygenase (COX)-1 and COX-2 enzymes. Grapiprant differs mechanistically from traditional NSAIDs by selectively inhibiting the EP_4_ receptor of prostaglandin E_2_. This targeted mechanism may reduce the risk of adverse events commonly associated with COX inhibition, such as vomiting, gastrointestinal ulceration, and acute kidney injury (152–155). Grapiprant is effective when treating dogs with OA (153, 156). In one clinical study, grapiprant was more effective than meloxicam to manage postoperative joint pain (157).

### Anti-nerve growth factor monoclonal antibodies (antiNGF mAbs)

Anti-nerve growth factor monoclonal antibodies (anti-NGF mAbs) are analgesic drugs that inhibit nerve growth factor (NGF) signaling. Bedinvetmab is an anti-NGF mAb that is FDA-approved for the control of OA pain in dogs, and is administered as a once-monthly subcutaneous injection. The clinical effects of anti-NGF mAbs have been described in several studies (4, 158, 159). Their analgesic efficacy in dogs with OA appears comparable with that of NSAIDs (160). The safety profile of anti-NGF mAbs differs from that of NSAIDs. Adverse events appear uncommon overall (160), but reports describe exacerbation of neurologic disease and the progression of joint degeneration in some cases (161–163). Several potentially different patterns of joint degeneration have been described, including rapid progression of joint degeneration, atypical periarticular bone remodeling, and extensive heterotopic periarticular mineralization, a process also named insidious anti-nerve growth factor-induced periarticular mineralization (163–165).

### Polysulfated glycosaminoglycans

Polysulfated glycosaminoglycans (PSGAGs) are disease-modifying agents that may influence cartilage metabolism and inflammation. A single PSGAG (Adequan) is approved by the FDA for the treatment of OA in dogs, and is administered as a series of 8 intramuscular injections given twice weekly. The beneficial effects of PSGAGs may be partly mediated by reductions in inflammatory mediators within the joint. In a pivotal study, growing dogs with hip dysplasia that received PSGAG between 2 and 10 months of age developed less cartilage damage and less joint laxity compared with untreated control dogs (166). Compounds related to PSGAG, such as pentosan polysulphate, have been used outside the United States for OA management. Beyond NSAIDs, anti-NGF mAbs, and PSGAG, the evidence supporting other pharmacologic classes used to manage OA pain in dogs is substantially more limited (4).

### Gabapentin and pregabalin

Gabapentinoids bind to the α2 δ-1 subunit of presynaptic voltage-gated calcium channels, reducing the release of excitatory neurotransmitters. These drugs are primarily intended to alleviate neuropathic pain. Gabapentin and pregabalin typically require administration two or three times daily to maintain therapeutic effects. Adverse effects may include transient sedation and ataxia. Commonly suggested doses are 10 to 20 mg/kg every 8 h for gabapentin (167) and 4 mg/kg every 12 h for pregabalin (168, 169). Optimal dosing in dogs remains uncertain, which may lead to underdosing in clinical practice. Gabapentin is frequently used as part of multimodal pain management strategies in dogs with chronic pain; however, evidence supporting its efficacy for OA-related pain is limited (128).

### Amantadine and ketamine

Amantadine and ketamine are *N*-methyl-D-aspartate (NMDA) receptor antagonists that act within the central nervous system. By inhibiting NMDA receptor activity in the spinal cord, NMDA receptor antagonism may help prevent or reverse central sensitization (“pain wind-up”) although they may not provide direct analgesic effects. In one clinical trial, amantadine administered once daily in combination with an NSAID increased activity levels in dogs with OA (170). Twice-daily dosing may provide greater benefit than once-daily administration (4, 167). Ketamine is sometimes used anecdotally to manage chronic pain when administered intermittently subcutaneously, at 0.5 mg/kg, or intravenously as a continuous rate infusion, typically at approximately 10 μg/kg/min for 4 h or longer.

### Opioids

Opioids are not commonly used for long-term management of chronic OA pain in dogs and are generally less effective for control of chronic pain than other analgesic classes. However, they may be useful for managing severe or breakthrough pain. Opioids are tightly regulated and commonly produce adverse effects, including sedation, nausea, and constipation. Increasing dietary fiber may help mitigate constipation in dogs receiving opioids chronically. The efficacy of orally administered opioids in dogs appears limited, possibly because of relatively poor oral bioavailability (167).

### Tramadol

Tramadol is an opioid-like analgesic with additional serotonergic and noradrenergic activity. In dogs, its analgesic effects are believed to result primarily from norepinephrine reuptake inhibition rather than classic opioid receptor activation. Dogs lack sufficient metabolic conversion of tramadol to its opioid-active metabolite (O-desmethyltramadol, M1), thus tramadol produces negligible μ-opioid receptor–mediated analgesic effects (171). When used alone, tramadol does not appear to provide meaningful relief of OA pain in dogs (117). However, some studies suggest that tramadol may provide additional benefit when combined with NSAIDs (172).

### Antidepressants

Tricyclic antidepressants such as amitriptyline have been considered for the management of chronic pain (169). Serotonin-norepinephrine reuptake inhibitors (SNRIs), including duloxetine, venlafaxine, desvenlafaxine, and milnacipran, are used to treat chronic pain in humans. However, information regarding their efficacy or safety in dogs with OA is currently lacking.

### Lidocaine

Lidocaine is occasionally administered as a continuous rate infusion in dogs with severe pain. In human medicine, perioperative lidocaine infusions reduce the risk of chronic pain development (173) and decrease opioid requirements (174).

### Glucocorticoids (corticosteroids)

Glucocorticoids are potent anti-inflammatory drugs but are associated with significant systemic adverse effects, particularly with long-term administration (150). Consequently, the use of systemic glucocorticoids for chronic OA management in dogs has become uncommon.

### Intra-articular injections

Unlike humans and horses (175, 176), the use of intra-articular (IA) injections to manage OA in dogs is relatively uncommon (177). IA injections include corticosteroids, hyaluronic acid, platelet rich plasma, autologous stem cells, bone marrow aspirate concentrate, stromal vascular fraction, polyacrylamide hydrogels (PAAG), and radioactive tin (tin-117m radiosynoviorthesis). IA injections in dogs are most often given under sedation. They require hair clipping and sterile preparation of the injection site. IA injections appear safe overall. Complications include a temporary increase in pain and lameness and septic arthritis. In one report evaluating the response to the IA injection of various products, one dog in five (18.4%) showed signs of pain after IA, with a median pain duration of 2 days (178). In that report, pain was mostly associated with the injection of stem cells.

#### Corticosteroids

Corticosteroids have a powerful anti-inflammatory action (150). Methylprednisolone acetate (up to 20 mg per joint) and triamcinolone acetonide (1 to 3 mg per joint) are used most often in dogs. Typical pain relief is visible after 12 to 24 h and can last up to 6 months (179). Side effects such as polyuria, polydipsia, or polyphagia are uncommon. However, corticosteroids appear to negatively impact cartilage and synovium, even after a single exposure (180). These adverse effects may not be present after triamcinolone exposure (181).

#### Hyaluronic acid (HA)

HA (sodium hyaluronate, hyaluronan) is a ubiquitous sugar glycosaminoglycan molecule present in all living organisms. HA binds to CD44 receptors on chondrocytes, inducing cellular proliferation and extracellular matrix production, stimulating synoviocytes to synthesize hyaluronan, an endogenous glycosaminoglycan, inhibiting inflammatory mediators, and exerting an anti-apoptotic effect on chondrocytes (182). Intra-articular HA aims to restore the physical properties of joint fluid to aid in joint lubrication, to decrease inflammation and degradation, and to help in cartilage repair (183). Several HA formulations are available for intra-articular injection. The use of high-molecular-weight HA is recommended because it more closely mimics naturally occurring HA. In a trial comparing high-molecular-weight HA to stem cell therapy, HA appeared more effective than stem cell therapy (184). However, the clinical benefits of IA HA appear modest overall, and IA HA is most often used in combination with other products.

#### Hydrogels

Several hydrogels are used clinically to manage OA in dogs. Collagen elastin hydrogel microparticles (CEHM) are manufactured from a purified self-assembling composition of collagen, elastin, and heparin. The peer-reviewed information documenting the efficacy of CEHM is limited. In one trial, the clinical response to CEHM and triamcinolone in dogs with OA did not differ from the response to triamcinolone alone (185). Polyacrylamide hydrogels provide viscosupplementation and act as a joint lubricant. Polyacrylamide hydrogels are available with several concentrations: the 2.5% polyacrylamide appears to be incorporated into synovium and 4% binds to cartilage (186, 187). The 2.5% polyacrylamide concentration appears safe and effective in human and equine OA patients (188, 189). In a dog owner survey (100 dogs, 150 injected joints), 10% of owners reported mild and self-limiting adverse events and 82% of owners considered their dog improved (190).

#### Regenerative medicine

Regenerative products (orthobiologics) include blood-derived and fat-derived products. Blood-derived products include platelet-rich plasma (PRP), autologous conditioned serum (ACS) or plasma (ACP), bone marrow aspirate, and bone marrow aspirate concentrate (BMAC). Fat-derived products include stromal vascular fraction (SVF) obtained from adipose tissue, either used directly or after culture and expansion. In the United States, the FDA classified orthobiologics as drugs (191). The most common orthobiologics used in dogs are autologous (patient-derived). PRP used alone or in combination with HA appears to be the most widely used orthobiologic product (192). Adipose or bone marrow stem cells have been used in dogs with OA and appear beneficial overall (193). Allogenic orthobiologic treatments are emerging, but evidence of their efficacy is currently lacking (194).

#### Radionuclide tin-117m

Radioactive tin-117m is approved by the FDA for management of synovitis in the dog elbow. The tin radionuclide emits low-energy electrons that promote necrosis of inflamed synovial cells. The clinical safety and the efficacy of radioactive tin-117m have been evaluated in small case series (195–197), and the treatment appears safe overall (198).

Potential cytotoxicity (i.e., chondrotoxicity) should be considered for all compounds injected into joints. Several local anesthetics (lidocaine, bupivacaine, ropivacaine) and corticosteroids (triamcinolone) are chondrotoxic (199). Chondrotoxicity appears to be mitigated when compounds are injected in combination with hyaluronic acid. Information about the chondrotoxicity of PRP is lacking (199). In one survey of 174 practitioners, 94% of respondents used IA therapy (200). The elbow was the most common joint injected. Platelet rich plasma and viscosupplements were the most commonly injected products.

### Pharmaceutical management: key points

NSAIDs remain the cornerstone of pharmacologic management for OA in dogs. Several adjunctive medications may provide additional benefit and generally have acceptable safety profiles, although their efficacy is less consistent than that of NSAIDs. A growing number of injectable therapies are available and may provide short-term clinical improvement, but evidence regarding their long-term effectiveness remains limited.

## Surgical interventions for management of OA in the adult dog

Surgery may be performed in joints affected by OA either with a therapeutic intent or as a salvage procedure. Therapeutic surgery aims to address the underlying cause of joint disease and may include removal of intra-articular osteochondral fragments, improvement of joint congruity or stability, or replacement of damaged cartilage with grafted cartilage. Salvage procedures include excision, replacement, or fusion of the affected joint.

### Hip joint

Hip dysplasia is the most common cause of OA in the hip joint (201) and is likely the most common orthopedic disorder in dogs. Other conditions that may lead to hip OA include Legg-Calvé-Perthes disease and fractures involving the femoral head growth plate (202). In dogs with hip dysplasia, OA typically progresses gradually over the course of a lifetime (8, 203), and excess body weight accelerates disease progression (203). Surgical procedures aimed at improving hip stability include juvenile pubic symphysiodesis, typically performed at approximately 16 weeks of age, and double pelvic osteotomy, generally performed around 6 months of age. Salvage procedures for advanced disease include femoral head and neck excision and total hip replacement (204). Evidence supporting the long-term efficacy of early therapeutic procedures for preventing OA progression is limited. In contrast, total hip replacement is associated with favorable long-term outcomes, but the surgery is complex and costly.

### Stifle joint

In the stifle joint, cranial cruciate ligament degeneration or rupture (CCLR) and patellar luxation are the most common causes of OA (11). Because owner satisfaction tends to be higher after surgical treatment compared with conservative management (205), CCLR is often treated surgically. Common surgical procedures include tibial osteotomy techniques and extracapsular stabilization using prosthetic material. Overall, outcomes following osteotomy procedures appear slightly superior to those achieved with extracapsular stabilization (206). Surgical correction is also frequently performed for patellar luxation (207). The goals of surgery are to restore proper patellar tracking within the trochlear groove and to realign the extensor mechanism of the stifle (i.e., the quadriceps muscle, patella, and patellar ligament). Surgical techniques are selected based on the severity of malalignment and may include trochlear deepening procedures, tibial tuberosity transposition, and soft tissue imbrication (207). Surgical treatment generally has a high success rate. However, because long-term studies evaluating OA progression after surgical correction are limited, it remains unclear whether OA progression continues following stabilization of the patella.

### Tarsal joint

OA of the tarsus is uncommon relative to the hip and elbow. It most often develops secondary to osteochondritis dissecans of the talus, intra-articular fractures, or from a traumatic luxation. Severe tarsal OA is typically managed with arthrodesis. Total ankle replacement and fitted exoprostheses are rarely used in dogs (208).

### Shoulder joint

OA of the shoulder is relatively common (209), although its underlying cause is often unclear. Osteochondritis dissecans of the humeral head is responsible for OA in a subset of dogs (202). Shoulder injuries are also reported to be the most common musculoskeletal injury in dogs participating in agility (210). These injuries may result in joint instability or, in severe cases, luxation. Surgical stabilization may be performed in dogs with significant shoulder instability (211). In cases of severe or chronic instability, shoulder arthrodesis may be considered as a salvage procedure.

### Elbow joint

OA of the elbow joint is common and most frequently develops secondary to elbow dysplasia (202), which typically affects the medial compartment of the joint (19). Surgical procedures intended to address elbow dysplasia include the arthroscopic or open removal of osteochondral fragments, joint resurfacing procedures, and osteotomies of the ulna or humerus. However, the scientific evidence supporting the efficacy of these procedures is limited (212). Total elbow replacement is rarely performed in dogs (213).

### Carpal joint

OA of the carpus is uncommon. It may develop following hyperextension injuries, intra-articular fractures, or immune-mediated disease. Severe instability and advanced carpal OA are most-commonly treated with carpal arthrodesis.

### Surgical management of OA: key points

OA most commonly affects the hip and elbow joints in dogs, largely due to the high prevalence of hip and elbow dysplasia. During the early stages of disease, a variety of surgical procedures may be performed to address the underlying cause or potentially slow OA progression, although evidence supporting the long-term efficacy of these interventions is limited.

## Integrating all approaches to manage OA in adult dogs

Epidemiological surveys suggest that approximately one in four adult dogs is affected by OA. Clinical signs vary widely, ranging from no detectable abnormalities to severe disability. However, many affected dogs experience persistent joint inflammation and pain. Effective management therefore aims to minimize the current impact of OA, slow disease progression, and optimize the dog's overall lifestyle. The physiologic impact of OA is primarily joint inflammation, which can be managed with medications, appropriate nutrition, and nutritional supplementation. The physical consequences of OA include pain and a potential loss of strength and fitness. These are addressed through pain management, physical activity, therapeutic exercise, and weight optimization. The functional impact of OA often manifests as reduced propulsion, decreased mobility, and diminished endurance, which may be improved through lifestyle modifications and ergonomic support.

Treatment plans should be individualized and influenced by the severity and chronicity of OA as well as by patient and owner factors (74). Although a wide range of therapeutic options is available, for many owners it is not feasible to implement all possible interventions. This underscores the importance of the veterinarian's role in counseling the owner when considering treatment options for particular patients and situations. It is important take owner goals and priorities into consideration, and streamline therapy whenever possible. Minimizing burden of treatment in terms of cost, time, effort, and risk may be beneficial in encouraging owners to pursue OA treatment fort their dog.

In most cases, OA is initially managed conservatively without surgery. The core components of conservative management include optimized nutrition and nutritional supplementation, analgesic medications, appropriate physical activity, and lifestyle modifications. These approaches can provide meaningful short-term relief and may also contribute to long-term joint health. Pharmacologic therapy is typically initiated with oral medications, most commonly NSAIDs, if they are well tolerated. The use of NSAIDs alone appears sufficient to improve joint pain and function in more than half of dogs with OA (151). When response to NSAIDs is incomplete, alternative therapeutics like anti-NGF mAbs and/or adjunctive oral medications are often considered, followed by intra-articular medications. Additional management strategies may also be considered when dogs cannot be adequately managed with weight optimization, nutritional supplementation, lifestyle modification, and pharmacologic therapy alone. These adjunctive options include electrophysical modalities, manual therapy, therapeutic exercise programs, and ergonomic support.

Among the available interventions, maintaining optimal body condition has the strongest evidence for long-term protection against OA progression (5, 6). Evidence also supports the benefits of regular physical activity and intramuscular administration of polysulfated glycosaminoglycans in slowing disease progression (166, 214). However, because OA in adult dogs is often longstanding at the time of diagnosis, interventions aimed at long-term joint protection may be less effective once structural joint changes are well established.

Sustained communication with the owner is essential for effective OA management. Standard assessment tools, such as the CBPI and LOAD questionnaires, can help quantify baseline disease impact, monitor response to therapy, and detect disease progression. Because many therapeutic interventions, particularly analgesic medications, should provide relatively rapid improvement in dogs experiencing OA-related pain, initial follow-up should occur within a few days of starting therapy. This follow-up helps ensure that the owner understands the treatment plan, that the therapy is effective and safe, and that it is practical to maintain over the coming months. If clinical signs are adequately controlled, reevaluation after approximately 3 months may be appropriate. If signs remain poorly controlled, earlier reassessment is warranted. Common causes of inadequate treatment response include persistent inflammation, chronic maladaptive pain, and physical deconditioning. Persistent pain may require adjustment of analgesic therapy or the addition of electrophysical modalities or manual therapy. Physical deconditioning may be addressed through targeted therapeutic exercise, including aquatic therapy (76), and additional ergonomic support. Periodic reevaluation, often on a quarterly basis, helps ensure that management remains effective as the disease progresses.

In summary, OA is common in adult dogs. Its management is complex but can be streamlined through a combination of optimized nutrition aimed at maintaining ideal body condition, appropriate nutritional supplementation, and effective pain control. NSAIDs remain the most predictably effective medications for managing OA-related pain and inflammation. Physical activity is beneficial and should be carefully tailored to minimize symptom flares. Ongoing communication with owners and regular reassessments are essential to maintaining effective long-term management of OA in affected dogs.
